# canSAR: update to the cancer translational research and drug discovery knowledgebase

**DOI:** 10.1093/nar/gkac1004

**Published:** 2022-11-29

**Authors:** Patrizio di Micco, Albert A Antolin, Costas Mitsopoulos, Eloy Villasclaras-Fernandez, Domenico Sanfelice, Daniela Dolciami, Pradeep Ramagiri, Ioan L Mica, Joseph E Tym, Philip W Gingrich, Huabin Hu, Paul Workman, Bissan Al-Lazikani

**Affiliations:** The Department of Genomic Medicine & The Institute of Data Science in Oncology, MD Anderson Cancer Center, Houston, TX, USA; The Department of Data Science, The Institute of Cancer Research, London, UK; Centre for Cancer Drug Discovery, The Division of Cancer Therapeutics, The Institute of Cancer Research, London, UK; Centre for Cancer Drug Discovery, The Division of Cancer Therapeutics, The Institute of Cancer Research, London, UK; The Department of Data Science, The Institute of Cancer Research, London, UK; The Department of Data Science, The Institute of Cancer Research, London, UK; Centre for Cancer Drug Discovery, The Division of Cancer Therapeutics, The Institute of Cancer Research, London, UK; The Department of Data Science, The Institute of Cancer Research, London, UK; Centre for Cancer Drug Discovery, The Division of Cancer Therapeutics, The Institute of Cancer Research, London, UK; The Department of Genomic Medicine & The Institute of Data Science in Oncology, MD Anderson Cancer Center, Houston, TX, USA; The Department of Data Science, The Institute of Cancer Research, London, UK; The Department of Data Science, The Institute of Cancer Research, London, UK; The Department of Genomic Medicine & The Institute of Data Science in Oncology, MD Anderson Cancer Center, Houston, TX, USA; Centre for Cancer Drug Discovery, The Division of Cancer Therapeutics, The Institute of Cancer Research, London, UK; Centre for Cancer Drug Discovery, The Division of Cancer Therapeutics, The Institute of Cancer Research, London, UK; The Department of Genomic Medicine & The Institute of Data Science in Oncology, MD Anderson Cancer Center, Houston, TX, USA

## Abstract

canSAR (https://cansar.ai) is the largest public cancer drug discovery and translational research knowledgebase. Now hosted in its new home at MD Anderson Cancer Center, canSAR integrates billions of experimental measurements from across molecular profiling, pharmacology, chemistry, structural and systems biology. Moreover, canSAR applies a unique suite of machine learning algorithms designed to inform drug discovery. Here, we describe the latest updates to the knowledgebase, including a focus on significant novel data. These include canSAR’s ligandability assessment of AlphaFold; mapping of fragment-based screening data; and new chemical bioactivity data for novel targets. We also describe enhancements to the data and interface.

## INTRODUCTION

canSAR (1–5) continues to be the largest public cancer drug discovery resource serving the international community with users from 400 countries and regions, both from academia and industry. canSAR has moved to its new home at MD Anderson Cancer Center and continues as an international collaboration between the authors. canSAR’s uniqueness comes from three key areas: (i) the full integration of data that are key for drug discovery (multi-omics, chemistry, pharmacology, systems biology, structural biology, and more); (ii) annotation and curation of these data to maximize their value for the drug discoverer; and (iii) a suite of unique to canSAR machine-learning algorithms, including comprehensive, multimodal, druggability assessment applied at scale. Although canSAR has a focus in oncology, most of its data, such as our AI-based druggability predictions, can be useful for any human disease. Areas of canSAR that focus on oncology include the disease pages, e.g https://cansar.ai/target/P15056/disease/skin-cancer (4) and the mutation annotation browser as well as the clinical trials browser, both of which were previously described (5). Meanwhile, the 3D structural analyses, all druggability assessments, chemical annotation, and interactome curation and visualizations apply to all human proteins without a specific focus on oncology.

In addition to growing the established data and making enhancements to the web interface to improve usability, the latest version of canSAR has significant new data that expands the offering for drug discovery. We have incorporated AlphaFold (6) structural prediction and XChem structural data (7), that for the first time enable us to estimate druggability across the entire human proteome. We have also abstracted and curated large-scale bioactivity data from publications outside the medicinal chemistry literature that were missing from public chemical databases. While modest in number, this effort expanded the target and compound coverage of canSAR to reach novel areas of biology not addressed in other public databases. We have also implemented a new chemical registration pipeline to better organize small molecules and their associated bioactivities, with particular emphasis on protein degraders.

## 3D STRUCTURE DATA AND ANALYSES

### canSAR 3D structure analysis and ligandability assessment

3D structural information provides fundamental insights into protein function and therefore represents an aid to selecting drug targets and accelerating drug discovery. canSAR analysis and 3D-ligandability (suitability for binding a drug-like ligand) assessment is updated weekly using 3D structural data from PDBe (8). We have previously described the method (2,9). In short, we devised a training set comprising 3D complexes of known drugs and their targets; as well as complexes of proteins bound to small molecule ligands that are Rule-of-Five compliant (10). We calculated 30 geometric and physicochemical properties for the cavities including volume, enclosure, polar and hydrophobic groups etc. We used these properties to train a decision-tree-based algorithm to classify druggable cavities against a background of cavities of unknown ligandability. As reported before (2,9). For every structure we analyze, canSAR scans for up to 10 cavities on each protein structure and assesses each cavity for compatibility of its geometric and physicochemical properties with small molecule drug binding using the machine learning algorithm. At the time of writing this update, canSAR contained >612,000 protein chains from >194,000 PDB structures and to date has identified and analyzed >5.4 million cavities of which >214,000 are predicted to be ligandable. canSAR also identifies ligandable cavities at the interfaces of biologically relevant macromolecular assemblies in the PDBe and has so far analyzed >651,000 cavities on >140,000 protein–protein interfaces and identified >91 000 ligandable interface cavities.

Additionally, canSAR evaluates ligandability of secondary, regulatory and allosteric ligandable sites. canSAR identified > 12,000 non-primary ligandable sites.

### New 3D structure data: AlphaFold and XChem

**Figure 1.**
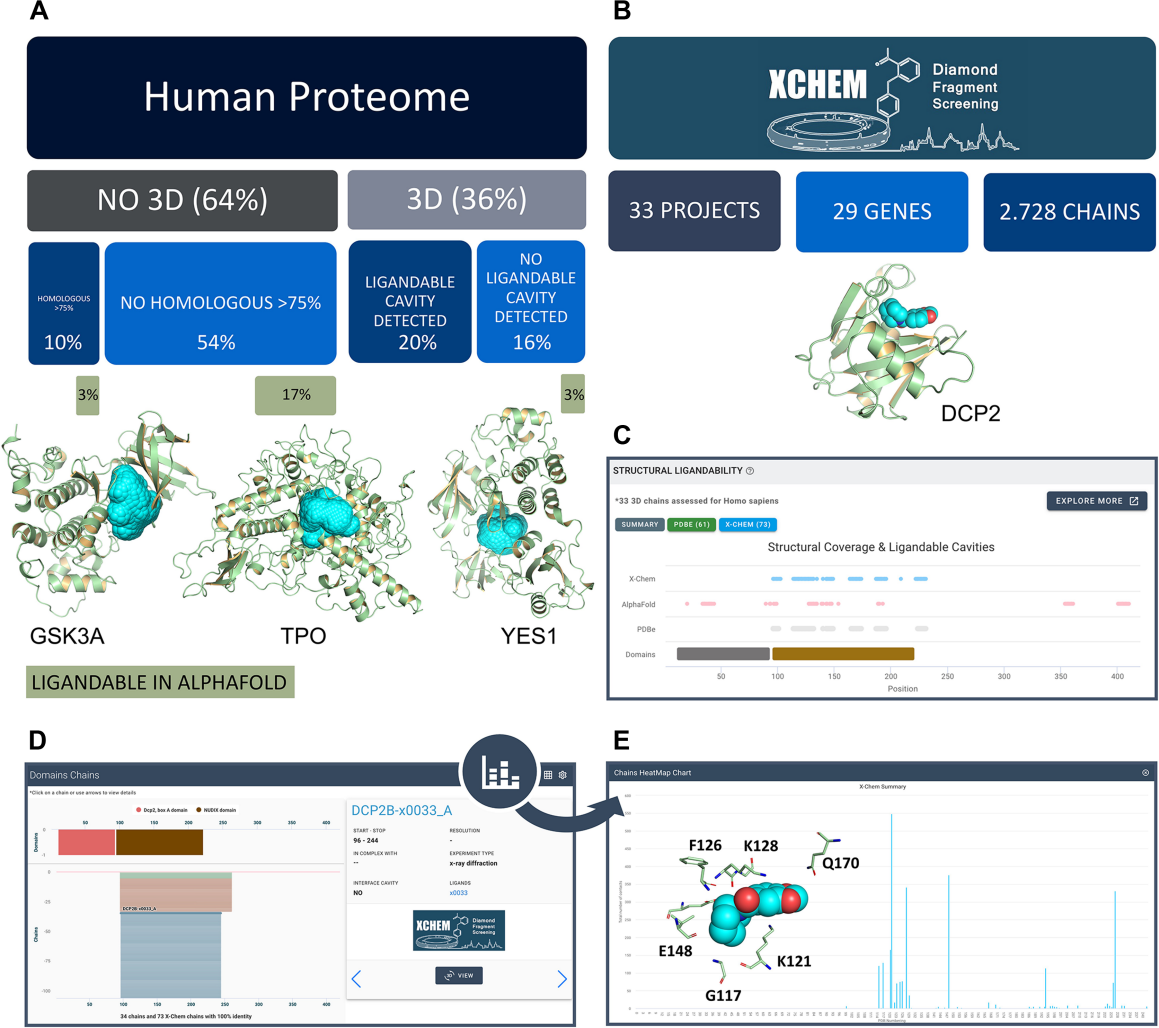
(**A**) Impact of AlphaFold models on uncovering new ligandable structures. 64% of the current human proteins currently lack experimentally-determined structures. Applying canSAR’s 3D-based structural assessment algorithm on the complete AlphaFold dataset, we were able to identify an additional 23% of human proteins potentially suitable for drug discovery. The well-known drug targets, GSK3A and TPO are two examples of proteins that lacked 3D structural coverage. Moreover, TPO does not have close homologous structures (≥75% of sequence identity). YES1 is an example of a drug target where canSAR did not previously report a ligandable pocket due to only partial 3D coverage. In this case, only the undruggable SH3 domain had experimentally-determined structures. AlphaFold models provide predicted structures for the other domains. canSAR analysis of the AlphaFold models identified druggable pockets on these new domains. (**B**) X-Chem statistics. 3D structures from 33 fragment screening projects have been added and assessed in canSAR. (**C**) A screenshot of the DCP2 protein synopsis page is shown. Residues that are part of the druggable cavities are shown in cyan dots and mapped on a 2D graph together with domains and other 3D structural data from AF and PDB. (**D**) The interactive, detailed view of domains, AlphaFold coverage as well as individual experimentally determined structures including those from X-Chem. (**E**) X-Chem advanced tool. The advanced tool is available from the Structural Ligandability section of canSAR and reports the overall number of interactions between residues and fragments, highlighting the residues interacting with fragments the most.

AlphaFold's success at predicting novel 3D protein structures has generated great hope for uncovering targets for therapeutic discovery that were previously hidden from view due to the lack of experimental structures (6). canSAR now includes the complete analysis and ligandability assessment of human AlphaFold 3D structures from the European Bioinformatics Institute (EMBL-EBI) (11). In Figure 1A, we report examples of novel ligandable proteins that have been uncovered through canSAR analysis of AlphaFold models. These proteins either had no experimental 3D structures determined, or were only partially structurally-characterized, and hence missing the ligandable domains. canSAR now provides ligandability assessment for 20,375 human proteins of which 13,052 have no experimentally-determined 3D-structures. 11,055 of these lack a close, structurally-determined, homologue (>75% of sequence identity). AlphaFold has revealed 3,979 novel ligandable human proteins of previously unknown structure. Care needs to be taken while utilizing some of these models to ensure that details are not anomalies of the AlphaFold predictive algorithms. 96% of the druggable cavities detected are formed in high confidence segments of the models (>50% of cavity residues have pLDDT ≥ 70; pLDDT is the AlphaFold measure of confidence in the prediction: predicted Local Distance Difference Test). In the canSAR structural viewer, users are able to color the AlphaFold model by pLDDT while examining the cavities. We recommend this in order that the user can get a detailed view of the confident versus non-confident parts of their cavity of interest.

Nonetheless, the capability afforded by enabling the canSAR analysis on AlphaFold models expands the reach of the drug discoverer to the rest of the human proteome not covered by the PDB. In addition, 3,296 proteins with known 3D structure previously lacked a ligandable cavity in canSAR due, for example, to only a partial structure being available which lacks the druggable domain. Using AlphaFold models, canSAR predicts ligandable cavities for 525 of these proteins. We have observed cases where the structural variation between the alpha-fold model and existing 3D structures for the same protein affected the local conformation of a particular cavity, sometimes altering its druggability score. We observe the same phenomenon when comparing the same cavity in different PDB structures of the same protein as described previously (2). Proteins structures are mobile and have spectra of different conformations which do impact the shape, availability of cavities and also may impact their druggability. For this reason, canSAR calculates the druggability for all cavities on every structure available rather than simply select a representative. We show all cavities and druggabilities on the canSAR interface to enable the user to judge the variation in their protein of interest, including the differences between the AlphaFold model and the experimental structures. Notwithstanding the need to take additional care, in total, AlphaFold increases the potential fraction of human proteins possessing at least one site that may be suitable for drug discovery from 19.8% to 41.8%.

We developed new visualizations and tools to summarize this new information in one place. Ligandability data for PDB, AlphaFold and XChem structures are easily accessible from synopsis pages on one page. A track-based view allows the user to see at summary level which parts of the protein have experimentally-determined structures; which have AlphaFold models; and where canSAR-identified ligandable cavities (Figure 1C). In this way, the drug discoverer can easily access the ligandable region of the protein.

All available structures as well as AlphaFold models are then represented on a more detailed, interactive view showing the span of each structure along the domains and the full protein sequence (Figure 1D). The user can click on the figure or use the carousel to navigate through each individual structure and examine its full detailed analysis.

Through a collaboration with XChem, canSAR now contains all unencumbered XChem 3D structures and small molecule hits identified through structure-based screening efforts at Diamond Light Source (7). canSAR now contains ligandability assessment for 2,728 chains from 33 screening projects for a total of 29 proteins (21 human proteins); all projects are listed in Supplementary Figure S1. In Figure 1E, we show the new XChem interaction tool we developed to easily access residue-fragment interaction data. Numbers of interactions for each residue are reported in the graph, highlighting the residues that interact with the fragments the most.

### Druggability beyond 3D structure

3D-structures provide an atomic-level view which informs drug discovery. However, it is not the sole source of information. canSAR provides several orthogonal assessments for the suitability of targets for therapeutic application and drug discovery. We provide ligand/chemistry-based assessment for 8,310 human targets using the chemical and bioactivity information within canSAR (4,9). For this ligand-based assessment, we group compounds that have shown activity against a specific protein and/or its close homologues. We then assess these compounds based on potency, chemical diversity and number of Rule-of-Five violations (4). We also calculate the network-based ‘target-likeness’ for 13,467 human targets. This uses network-based properties of the human interactome to train an ensemble of machine-learning models to recognize cancer drug targets and assess novel proteins for the similarity of their network behavior to these drug targets (13). Finally, we tag proteins that are extra-cellular or have sufficient extra-cellular regions that would be easily available for antibody therapeutics. We find that at least 3,586 targets would be accessible to such biotherapeutics. This information continues to be provided for each human target on the Molecular Synopsis page of canSAR (e.g. https://cansar.ai/target/P00533/synopsis/ligandability)

## BETTER ANNOTATION AND ORGANIZATION OF BIOLOGICALLY ACTIVE SMALL MOLECULES

A key challenge for the drug discoverer is analyzing data relating to the same compound from different sources. Differences in chemical representation of the same compound (e.g. tautomers) can result in separation of data that should be assigned to the same compound (14). In other cases, scientists would benefit from data belonging to related compounds (e.g. racemates) but these data can be easily missed in most resources. We have recently published canSARchem (14), an innovative canSAR chemical registration pipeline that standardizes, corrects, and groups related compounds in biologically meaningful, consistent hierarchies. canSAR’s data are now organized using this pipeline, thereby allowing the drug discoverer to easily identify and link all biological data relating to a compound or family of compounds.

**Figure 2.**
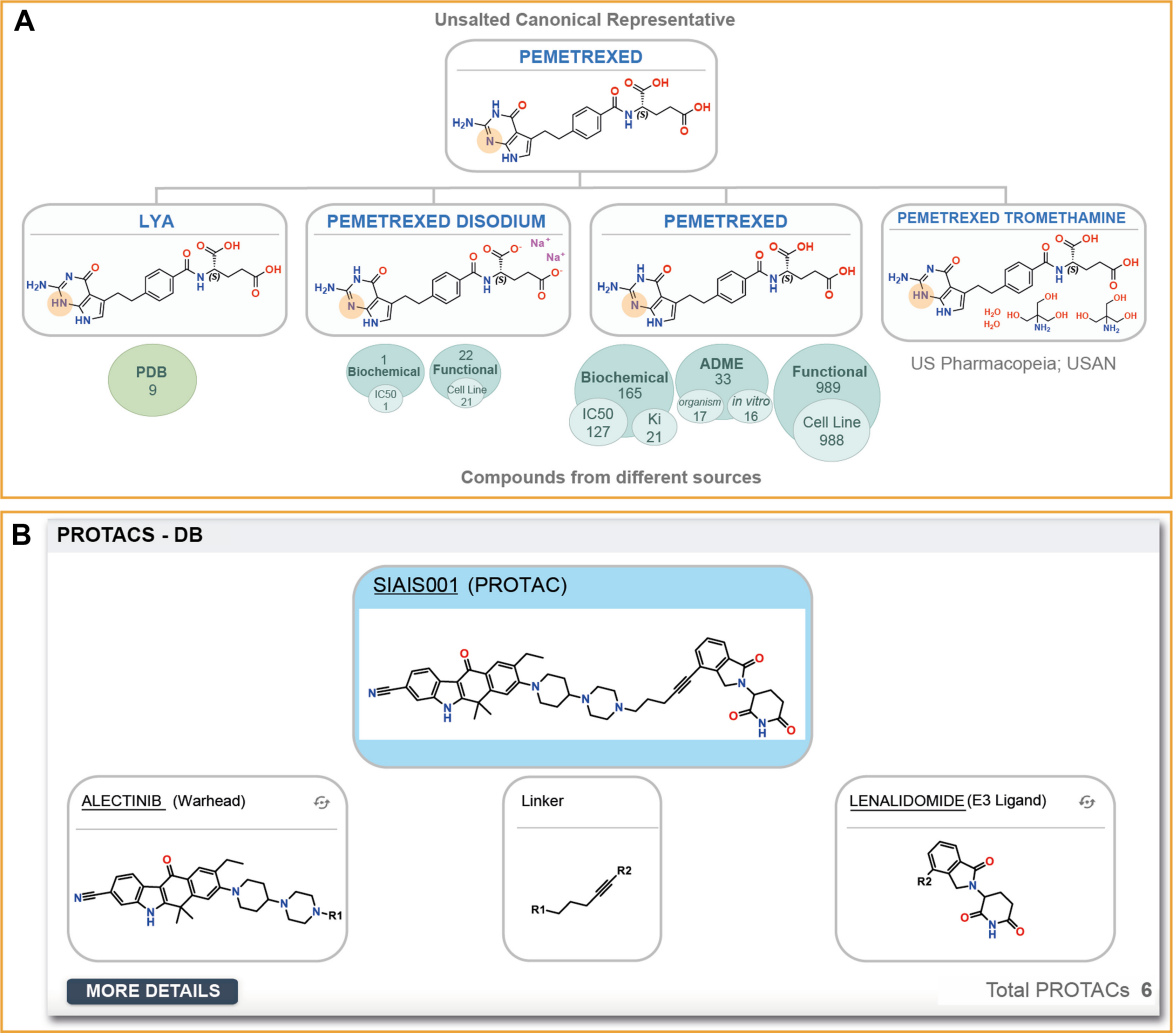
(**A**) Pemetrexed compound family at the unsalted canonical representative level. Includes five different registered compounds that represent two tautomers and three salted forms. As can be observed, three of the compounds are associated with different bioactivity data that might be important to consider. For example, LYA is co-crystalized in nine PDB structures whilst pemetrexed has associated Biochemical, ADME and Functional data. Through the canSAR compound family, the user is readily alerted to these different compound forms and can explore all the data associated with each one to gain a comprehensive perspective of the known bioactivity of pemetrexed. (**B**) PROTAC hierarchy of the compound SIAIS001. As can be observed, the PROTAC chemical structure is displayed at the top (in blue), whilst the substructures of its warhead, linker and E3 ligand are displayed at the bottom. The user is also alerted to the fact that there are in total six PROTACs with the same warhead and E3 ligand, which can be explored by clicking the ‘MORE DETAILS’ button.

For example, the pyrrolopyrimidine-based antifolate chemotherapy drug pemetrexed has been deposited in the PDBe (8) and ChEMBL (15) in two different tautomeric forms that correspond to two distinct entries (Figure 2A). This makes it easy for a non-expert user to miss the fact that the co-crystal structure of pemetrexed with eight different proteins is available in the PDBe (Ligand HETAOM ID: LYA) and also has biochemical and functional data deposited in ChEMBL. Through canSAR, the user can easily identify that pemetrexed is part of a compound family comprising three different salted forms and two tautomers (Figure 2A), and is prompted with the whole structural, biochemical and functional data to make an informed analysis. We have recently shown that FDA-approved, small molecule drugs present an enrichment in tautomeric forms as compared to other compounds (14); this facilitated identification of data related to different tautomers is particularly important for canSAR’s translational research and drug discovery users.

Bifunctional protein degraders (PROTACs) and molecular glue degraders are important emerging classes of bioactive small molecules (16). Accordingly, we have invested significant efforts in their registration and organized them efficiently to help the user make the most of these data. We now incorporate PROTACs and molecular glues from The Chemical Probes Portal (12) and PROTAC-DB (17). We have organized the PROTACs into a hierarchy, highlighting the corresponding warhead, E3 ligand and linker. Overall, canSAR so far includes 2596 PROTACs that use 359 warheads, 89 E3 ligands and 1085 linkers. Figure 2B shows the hierarchy of the ALK degrader SIAIS001, which is rated positively on the Chemical Probes Portal (chemicalprobes.org). We have adapted the structures of warheads and E3 ligands to clearly mark the substitution point (R1 and R2), but we enable users to switch the image to see the original chemical structure (without ‘R’). Linkers are not registered in canSAR as compounds since they are substructures and therefore not necessarily compounds that exist independently. The user is presented with the information on how many compounds share the same warhead and E3 ligand but a different linker and can deep dive into these data to explore the different linkers used. In future, we will improve the integration of PROTAC chemical structures with associated proteomics data, and develop tools to facilitate the analysis of how different linkers, warheads and E3 ligands influence bioactivities and other properties.

## NEW BIOACTIVITY DATA

**Figure 3.**
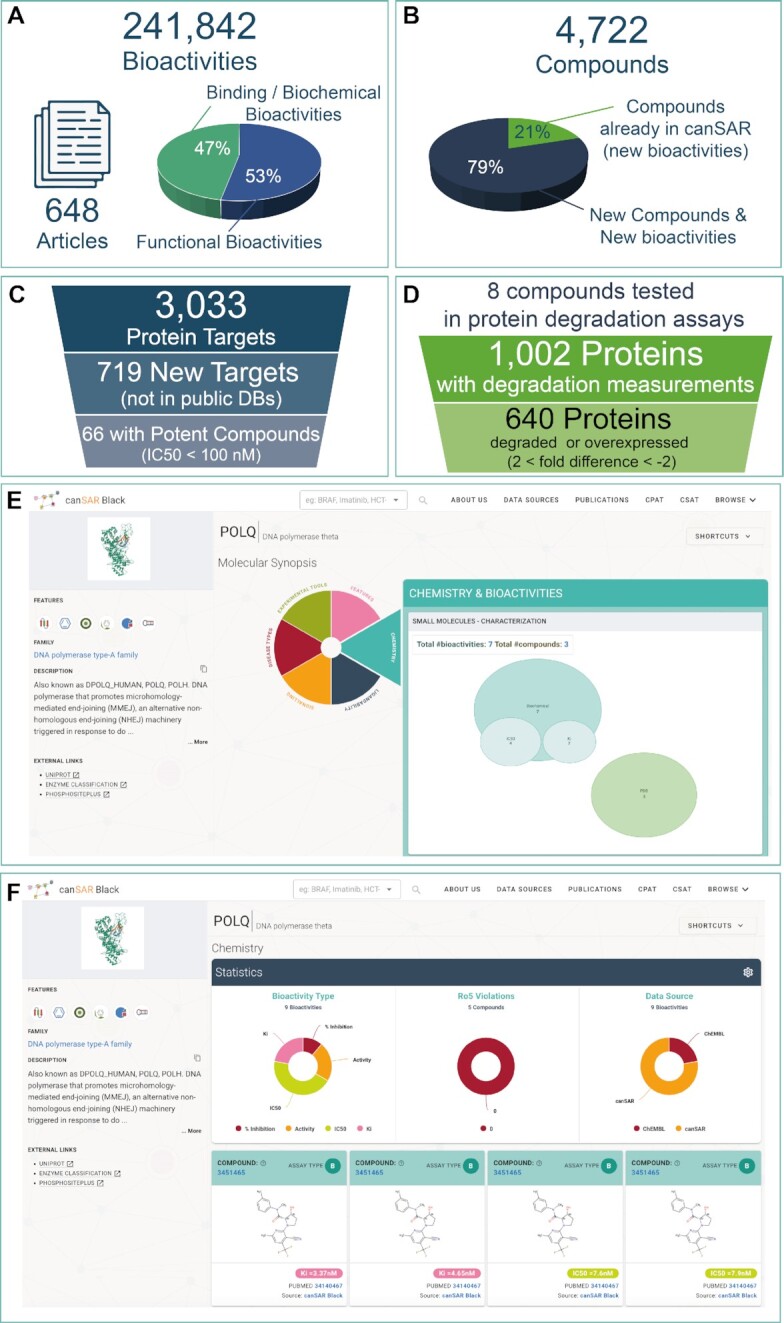
Summary of the data abstracted from non-medicinal chemistry journals. (**A**) A schematic summarizes the statistics of the abstracted data, including the number of articles abstracted and the corresponding bioactivity data points, distinguishing between target and cellular bioactivities. (**B**) Number of compounds that were abstracted and pie chart illustrating that most of them are not present in other public resources. (**C**) A funnel-type summary of the number of targets that were abstracted in the target bioactivities, how many of them are not present in other public resources and how many of them are inhibited more potently than 100 nM. (**D**) Funnel-type scheme of the number of compounds and targets that have degradation data, highlighting the targets that are degraded or overexpressed above a 2-fold threshold. (**E**) Snapshot of the landing page of POLQ in canSAR to illustrate how these unique data are displayed on the canSAR website. (**F**) Detail of some of the abstracted POLQ inhibitors and their bioactivity assay values displayed in the canSAR website.

canSAR continues to integrate chemistry and bioactivity data from valuable major public resources such as ChEMBL (15), BindingDB (18), The Chemical Probes Portal (12), etc. However, typically, these resources focus on core medicinal chemistry journals and, as a result, miss many new targets and compounds that span areas of biology not covered by these databases. This is particularly important in translational research as many important chemical probes and clinical candidates are published in journals outside the medicinal chemistry literature and, therefore, very valuable selectivity data are missing from public databases (19). We have invested in abstracting data focused on these areas that are missing from other public databases. As a result, canSAR now contains >240 000 new pharmacological measurements, not available anywhere else in the public domain, derived from 648 publications (Figure 3A). A total of 47% of these data involve binding or biochemical assay values with the rest being data from functional assays such as cellular growth inhibition (Figure 3A). Overall, our curation of data from the non-medicinal chemistry journals has added >3000 high value novel bioactive and experimentally verified small molecules not available in public databases elsewhere (Figure 3B) and increased the number of chemically characterized targets to an additional 719 human proteins not previously available in public resources, 66 of which have a small molecule displaying binding activity more potent than 100 nM (Figure 3C). We have also incorporated degradation data with a significance threshold of 2-fold for an additional 640 proteins (Figure 3D). As an example of the value of these data, the important cancer drug target POLQ (Figure 3E), that has no bioactive small molecules reported in other databases, has seven bioactivity values in canSAR (Figure 3E) including the potent, low-nanomolar, clinical candidate ART558 (canSAR3451465) that has been recently published (20). These data confirm that this protein has been already drugged and potent compounds are available. In addition, we incorporate functional measurements for >1000 human proteins degraded by PROTACs. Overall, the new curated bioactivity data significantly expand the coverage of canSAR for the benefit of our users.

## IMPROVING THE USABILITY

The inclusion of new data types and the continuous growth of data in canSAR has been accompanied by several improvements in usability, benefiting from feedback from the canSAR User Group and the broader scientific community.

The new PROTAC interface (Figure 2B), is fully integrated with the existing compound hierarchies derived by canSARchem, and further provides interactive visualization of PROTACs sharing the same warhead, E3 ligand or linker. Where necessary, user interfaces have been redesigned. For example, the introduction of AlphaFold and XChem data has led to the new structural ligandability display (Figure 1B), which overlays the new assessments on the existing PDB ligandable cavities and 3D structural coverage. Selecting a specific data source provides in-place frequency visualizations of the ligandable cavity amino acid residues. Furthermore, filters by species and sequence homology allow fine-tuning of the available data to the user needs and all ligandability assessments are now downloadable in PyMol-compatible format.

Several small improvements in the existing canSAR pages collectively enhance the user experience: in pages pertaining to molecular alterations in disease, filters allow grouping of the available TCGA and ICGC studies by primary site and cancer type, whereas cancer cell line model lineage has been simplified to improve copy number assessments for a given molecular target. Finally, broad-spectrum usability improvements include slimlining the interface by displaying only modules and menus where data / analyses are available, providing quick links to disease, molecular alterations and chemistry pages via pull-down menus and the introduction of search banners in all relevant pages to effortlessly explore data of interest.

Overall, the new canSAR front-end delivers a significant usability overhaul, whilst maintaining the navigation philosophy and the look and feel that users have been accustomed to.

## CONCLUSION

canSAR (https://cansar.ai) continues to support cancer translational research and drug discovery efforts internationally through the integration of multidisciplinary data relevant to drug discovery, and through the application of unique machine-learning algorithms to these data. The enhancements to the data and interface enable researchers to derive new value from resources such as AlphaFold, XChem, and more.

## DATA AVAILABILITY

canSAR is freely available at https://cansar.ai.

## Supplementary Material

gkac1004_Supplemental_File

## Notes

Present address: Daniela Dolciami, Benevolent AI, London, UK.
