# Supplementary material for: canSAR: update to the cancer translational research and drug discovery knowledgebase
Source: Nucleic Acids Res. 2022 Nov 29;51(D1):D1212–9. doi: 10.1093/nar/gkac1004 (PMC9825411; doi:10.1093/nar/gkac1004)
Supplement: gkac1004_Supplemental_File [file gkac1004_supplemental_file.pdf]

SUPPLEMENTARY

Fig. S1. List of XCHEM projects included in canSAR

Uniprot ID	*gene name*	*XCHEM ID*	*N. of chains*	*With Fragment*
B2RID1	dpp11	PGN_RS02895PGA	28	28
O95696	BRD1	BRD1A	14	14
P0DTD1-Nsp13	Hel	nsp13	255	205
P22188	murE	MUREECA	18	18
		MUREECOLI	2	2
Q460N5	PARP14	PARP14A	18	18
Q8IU60	DCP2	DCP2B	73	73
Q92835	INPP5D	INPP5DA	281	190
Q9Y2J2	EPB41L3	EPB41L3A	204	153
O15178	TBXT	TBXTA	49	49
P0C024	NUDT7	NUDT7A	11	11

		NUDT7ACRUDE	257	257
P0DTD1-Nsp15	Nsp15	NSP15_B	24	12
P24821	TNC	TNCA	17	17
Q5T0W9	FAM83B	FAM83B	13	13
Q8IU85	CAMK1D	CAMK1DA	19	19
Q9NZJ9	NUDT4	NUDT4A	12	12
O43809	NUDT21	NUDT21A	62	62
P0DTC1-Nsp3	Nsp3	Mac1	927	603
		mArh	235	235
P14489	bla	OXA10OTA	20	20
Q04771	ACVR1	ACVR1A	4	4
Q6PJP8	DCLRE1A	DCLRE1AA	27	27
Q8WVM7	STAG1	STAG1A	38	38
Q9UJM8	HAO1	HAO1A	25	25
O60229	KALRN	XX02KALRNA	24	24

P0DTC1-Nsp5	3CL-PRO	Mpro	586	292
P15379	Cd44	CD44MMA	46	23
Q12830	BPTF	FALZA	8	8
Q6PL18	ATAD2	ATAD	5	5
		ATAD2A	2	2
Q8WWQ0	PHIP	PHIPA	147	49
Q9UKK9	NUDT5	NUDT5A	220	220
		APO (no fragments)		
P14900	murD	MURD	4	-
Q9NZJ9	NUDT4	NUDT4	6	-
P18031	PTPN1	PTP1B	127	-
Q962Y6	TGR	SMTGR	23	-
		smTGRNEW	8	-
			3839	2728
